# Sensor-Based Vibration Signal Feature Extraction Using an Improved Composite Dictionary Matching Pursuit Algorithm

**DOI:** 10.3390/s140916715

**Published:** 2014-09-09

**Authors:** Lingli Cui, Na Wu, Wenjing Wang, Chenhui Kang

**Affiliations:** 1 Key Laboratory of Advanced Manufacturing Technology, Beijing University of Technology, Chaoyang District, Beijing 100124, China; E-Mails: adinala@emails.bjut.edu.cn (N.W.); kavie0331@sina.com (C.K.); 2 School of Mechanical, Electrical and Control Engineering, Beijing Jiaotong University, No.3 Shangyuancun, Haidian District, Beijing 100044, China

**Keywords:** composite dictionary single-atom matching, termination condition of iteration, fault diagnosis, modulation dictionary, sensor-based vibration signals

## Abstract

This paper presents a new method for a composite dictionary matching pursuit algorithm, which is applied to vibration sensor signal feature extraction and fault diagnosis of a gearbox. Three advantages are highlighted in the new method. First, the composite dictionary in the algorithm has been changed from multi-atom matching to single-atom matching. Compared to non-composite dictionary single-atom matching, the original composite dictionary multi-atom matching pursuit (CD-MaMP) algorithm can achieve noise reduction in the reconstruction stage, but it cannot dramatically reduce the computational cost and improve the efficiency in the decomposition stage. Therefore, the optimized composite dictionary single-atom matching algorithm (CD-SaMP) is proposed. Second, the termination condition of iteration based on the attenuation coefficient is put forward to improve the sparsity and efficiency of the algorithm, which adjusts the parameters of the termination condition constantly in the process of decomposition to avoid noise. Third, composite dictionaries are enriched with the modulation dictionary, which is one of the important structural characteristics of gear fault signals. Meanwhile, the termination condition of iteration settings, sub-feature dictionary selections and operation efficiency between CD-MaMP and CD-SaMP are discussed, aiming at gear simulation vibration signals with noise. The simulation sensor-based vibration signal results show that the termination condition of iteration based on the attenuation coefficient enhances decomposition sparsity greatly and achieves a good effect of noise reduction. Furthermore, the modulation dictionary achieves a better matching effect compared to the Fourier dictionary, and CD-SaMP has a great advantage of sparsity and efficiency compared with the CD-MaMP. The sensor-based vibration signals measured from practical engineering gearbox analyses have further shown that the CD-SaMP decomposition and reconstruction algorithm is feasible and effective.

## 1. Introduction

Gears are widely used in transmission systems of industry production lines. It is directly related to the normal operation of production efficiency and equipment safety. However, the nonlinear and non-stationarity of the gear vibration signal itself with noise makes it more difficult to diagnose. For this kind of signal, many time-frequency analysis methods based on vibration signals have been developed, which include the Wigner–Ville distribution [1,2], wavelet transform [3,4], Hilbert–Huang transform [5] and empirical mode decomposition and its extension [6–8]. However, the single basis function of general time-frequency analysis methods limits the applying of the effect with respect to adaptability and flexibility. In this regard, lots of scholars have explored a new way for signal representation, called the over-complete redundant function, to replace the traditional basis function. The over-complete redundant function was named as the atom dictionary, which is made up of a series of atoms with a similar structure, but with different parameters. Appropriate atoms were picked to present a signal in linear combination; this method was called sparse approximation [9]. In 1993, Mallat and Zhang proposed matching pursuit (MP) [10,11], which achieves a sparse expression of the signal. Besides, researchers did a lot of work to optimize the MP algorithm and to expand its fields of application.

The classic matching pursuit (MP) matching atoms are too single, and the computation is too large, which restricts the application of the algorithm. Therefore, research on the MP algorithm is concentrated on the atom dictionary construction and the fast algorithm in atomic decomposition. The common function model to build the dictionary includes the Fourier function, wavelet function, Gabor function and wavelet packet function [12–17]. In 2009, Wang [18] proposed an atom dictionary built of a series of characteristic waveforms to recognize the fault patterns of a rolling bearing. In 2011, [19] proposed a composite dictionary to extract the gear fault feature, the new dictionary on the basis of the composition and the structure of the fault signal, achieving a good effect. The work in [20,21] proposed a dictionary with the same waveform, but a different time-delay parameter, and the inner product operation between the signal and set could change to the correlation function method. The work in [22,23] combined the genetic algorithm with MP to improve the accuracy and to reduce the computation. However, the intelligent algorithm is too random and needs a huge amount of computation. The work in [11] proposed an adaptive dictionary MP method, which studied each key parameter in the dictionary model and its influence on the analysis results first, then established the adaptive impulse dictionary by changing characteristic parameters progressively. The work in [19] proposed a piecewise interception in the decomposition process to improve operation efficiency.

Above all, this paper proposes a new method for the analysis of gear fault, named the composite dictionary single-atom matching pursuit (CD-SaMP) decomposition and reconstruction algorithm, with a similar idea as in [19], which constructed a composite dictionary, applied a genetic algorithm to select the best matching atoms in the dictionaries, reduced the signal noise dependence on the threshold proportion of the distribution, and combined sectional interception to improve the operating efficiency. However, this paper optimizes this a lot: First, the optimization algorithm adds the modulation dictionary in the composite dictionary in order to enrich the dictionary, while the original composite dictionary was only made up of the Fourier dictionary and the impulse time-frequency dictionary. Second, the threshold de-noising method influences the result a lot, because the choice of the threshold is too random. More importantly, the original multi-atom matching was just to achieve a noise reduction in the reconstruction stage, but could not really decrease the calculation quantity and improve the efficiency at the decomposition stage, which was the fundamental reason leading to low efficiency. Although piecewise interception way help a lot, it is still not complete. Therefore, this paper proposes a CD-SaMP algorithm, which optimizes the termination condition of iteration based on the attenuation coefficient, reduces the introduction of noise in the process of decomposition and improves the operating efficiency of the algorithm and the sparsity in the decomposition.

The paper is organized as follows: Sections 2 and 3 address the specific decomposition and reconstruction algorithm of CD-SaMP. Section 4 modifies the type of dictionary and the termination condition of iteration. Section 5 presents the simulation signal analysis results and compares the superiority with the original multi-atom method. The algorithm is validated through an application example of sensor-based vibration signals measured from a practical engineering gearbox in Section 6. Finally, Section 7 concludes the paper with some remarks about possible future work.

## 2. CD-SaMP Decomposition and Reconstruction Algorithm

Different feature atom libraries are combined to form the composite dictionary. Each iteration process of the matching pursuit algorithm would seek a best matching atom to make its matching coefficient maximum, and the sub-feature atom library from which it comes was recorded. After decomposition, the matching atoms of the various orders and the matching coefficients are obtained. Reconstruction is an inverse process of decomposition. To reconstruct all of the atoms would yield the reconstructed signal. If only the atoms from a certain feature atom library are reconstructed, a certain feature component could be obtained. The above algorithm is called the CD-SaMP decomposition and reconstruction algorithm. The specific steps of the decomposition algorithm are as follows:

(1) The parametric model function is used to construct the sub-feature atom library *ϕ_i_*; different *ϕ_i_* are combined into a composite dictionary *D* = {*ϕ_i_*}.
(2) Initializing the residual signal *r*_0_ = *x* (*t*).
(3) Finding the atom *d_m_* that best matches the residual signal *r_m_* (*M* = *0* … *M* − *1*, *M* is the number of iteration) in the composite dictionary *D*.
(4) Calculating the matching coefficient *c_m_* and recording the sub-feature library *ϕ*_i_ from which the matching atoms come, that is *d_m_*∈*ϕ_i_* and *d_m_* is denoted as *d_mi_*; *c_m_* is denoted as *c_mi_*.
(5) Solving projection *p_m_* = *c_m_d_m_* and updating the residual signal *r_m_* + 1 = *r_m_* − *p_m_*.
  (1) $$p_{m}=\sum_{i=1}^{I}c_{mi}d_{mi}$$
(6) Repeating Steps (3)–(5), until the termination condition is met.
(7) Matching atom *d_mi_* and matching coefficient of *c_mi_* of each order are obtained at the end of the decomposition.

The calculation formula of the reconstruction algorithm is expressed as follows:

(2) $$\tilde{x}=\sum_{m=1}^{M}c_{m}d_{m}$$

If reconstruction only comes from the signal component of one certain sub-feature atom library, then the reconstruction formula is expressed as follows:

(3) $$\tilde{x_{i}}=\sum_{m=1}^{M}c_{mi}d_{mi}$$

## 3. CD-SaMP for Gear Fault

A key step of the matching pursuit algorithm is to choose the appropriate dictionary. This paper mainly aims at the gear signal. The frequency components of the gear signal are complex, including the meshing frequency, self-vibration frequency and the side frequency caused by the modulation of the fault. In order to match the shock and transient vibration characteristics of the fault gear, the Fourier dictionary, the impulse time-frequency dictionary [24] and the modulation dictionary are constructed using the method of the parameterized function model, and the Fourier dictionary and the modulation dictionary are combined with the impulse time-frequency dictionary to form a composite dictionary independently. The construction method is described in detail as follows.

The primitive function of the Fourier dictionary is a sine function; the model can be expressed as the following equation:

(4) $$g_{\text{fou}}(f,\gamma )=K_{\text{fou}}\sin(2\pi ft+\gamma )$$

where *f* is the frequency parameter (Hz), *γ* is the phase parameter and *K_fou_* is the normalized coefficient.

The primitive function of the Fourier dictionary is a sine function impulse time-frequency dictionary referring to the exponential decay function, *i.e.*,

(5) $$g_{\text{imp}}(p,u,f,\Phi )=\left\{ \begin{array}{l} K_{\text{imp}}e^{-p(t-u)}\sin2\pi f(t-\Phi ),t\geq u\\ 0,t<u \end{array}\right.$$

where *p* is the damping characteristic of the impulse response, *u* is the initial time when an impulse response event occurs (*s*), *f* is the damped natural frequency of the system (Hz), Φ is the phase deviation and *K_imp_* is the normalized coefficient.

The primitive function of the modulation dictionary is an amplitude modulation function, *i.e.*,

(6) $$\phi_{\mathrm{mod}}(f_{1},f_{2})=K_{\mathrm{mod}}(1+\cos2\pi f_{1}t)\cos2\pi f_{2}t$$

where *f*_1_ is the low-frequency modulation frequency (Hz), *f*_2_ is the high-frequency modulation frequency and *K*_mod_ is the normalized coefficient.

Assign to each parameter of the function model a certain range. The function model will be substituted by a series of parameters to obtain an atom, and the dictionary is formed by these atoms. To improve the computational efficiency, the genetic algorithm (GA) is used to choose the best matching atom in each iteration of the matching pursuit. The genetic algorithm is an iterative adaptive probabilistic search algorithm based on the principle of natural selection and natural genetic mechanism and does better at achieving the goal of optimizing the advantages than other traditional optimization algorithm. As mentioned in [19], joint coding was first performed on all of the parameter groups needed for constructing characteristic atom dictionaries, to produce randomly an initial population with a certain scale *N* in this algorithm. Each series of parameters is considered as an individual, and crossing and mutation are conducted according to a certain probability. Then, calculate the fitness value of each individual and choose the maximum fitness individuals as the best ones for the next generation directly. Choose *N* − 1 individuals from the parent generation with the random iteration method to the next generation; all of these individuals form a new population. Like the generational populations, the new population repeats the crossing, mutation, fitness calculation, selection and other operations to continuously evolve, until the evolution generations reach a preset value. Finally, select an individual with the maximum fitness from the optimal ones in each generation as the optimal parameter group, and decode them to substitute into the primitive function to form the optimal matching atom.

## 4. Gear Simulation Signal Analysis

### 4.1. Simulation Signal Construction

As the CD-SaMP is an improved algorithm of the composite dictionary multi-atom matching pursuit (CD-MaMP), the parameters of the simulation signal are chosen to be the same as those in [19] in order to get an obvious comparison between the two methods. Therefore, in this paper, the vibration signal model for gears with cracking fault is also the function mentioned in [25]:

(7) $$y(t)=\sum_{m=0}^{M}A_{m}[1+{\tilde{a}}_{m}(t)]\cos\{2\pi f_{m}t+\beta_{m}+{\tilde{b}}_{m}(t)\}+d(t)\cos(2\pi f_{r}t+\theta_{r})$$

**Figure 1.**
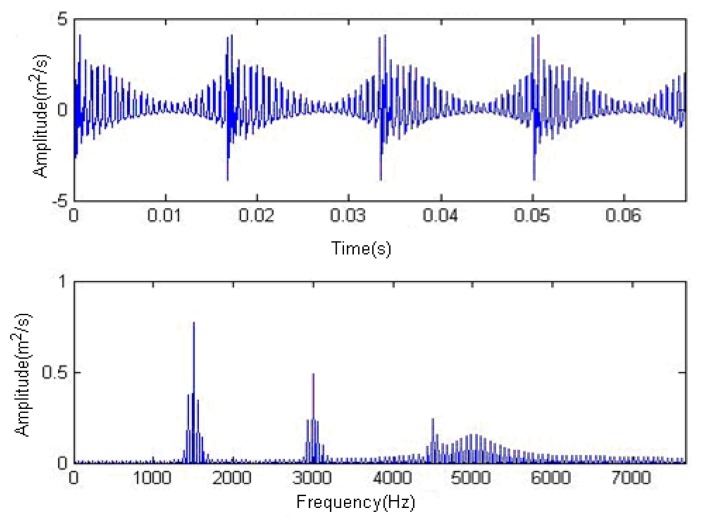
Signal waveform and frequency spectrum.

**Figure 2.**
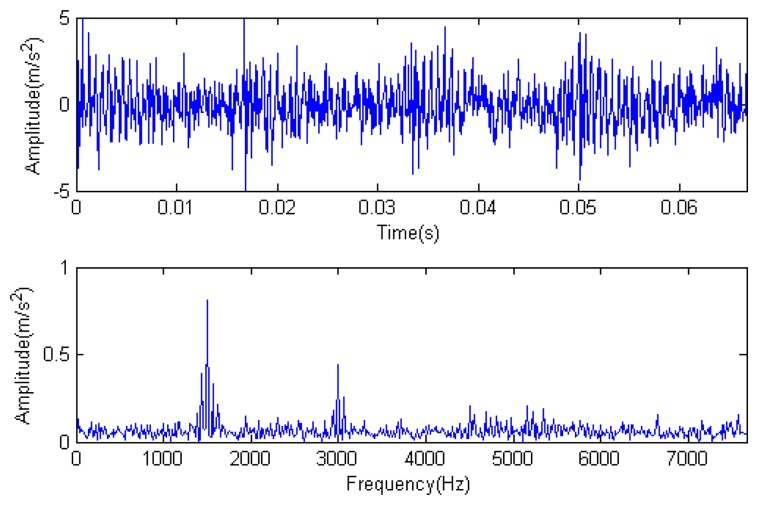
Signal waveform and frequency spectrum with noise.

The meaning of the each parameter in Equation (7) can be found in the original literature [19]. The sampling point is set to be 1024 points, and the time-domain waveform and spectra upon the two rounds of gear rotation are shown in Figure 1. From the frequency spectrogram in Figure 1, the meshing frequencies of the three orders (1.5, 3 and 4.5 kHz), their modulation sidebands and the impulse response bands in the vicinity of 5 kHz can be clearly found. To approach the real fault signals better, random noise in the standard normal distribution is introduced. The signal-to-noise ratio (*SNR*) after noising is −0.7339 dB (the formula for calculating *SNR* is shown in Equation (8)). The waveform and the frequency spectra of the signal with noise are shown in Figure 2. From the frequency spectrogram in Figure 2, the system resonance band caused by the fault shock after noising is basically overwhelmed by the noise.

(8) $$SNR=20\log_{10}(v_{s}/v_{n})$$

where *v_s_* and *v_n_* are the effective values of the primary simulation signal and the noise, respectively.

### 4.2. Termination Condition of Iteration Selection

#### 4.2.1. Traditional Termination Condition of Iteration

**Figure 3.**
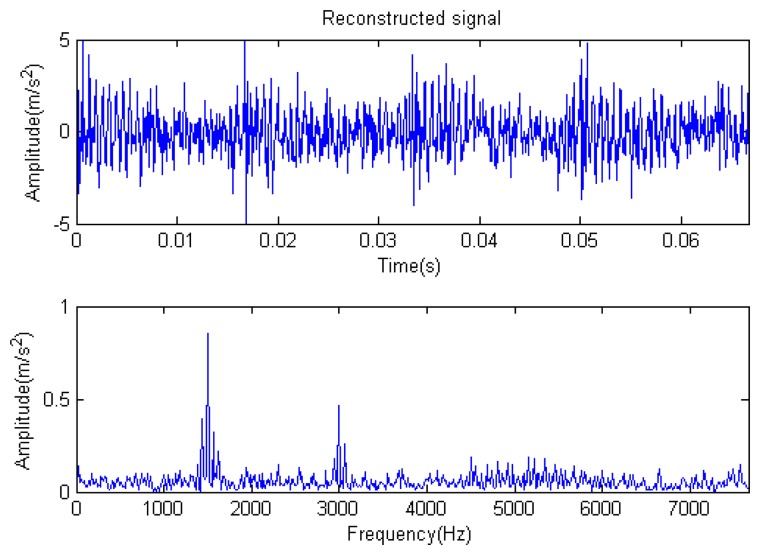
Reconstructed signal.

**Figure 4.**
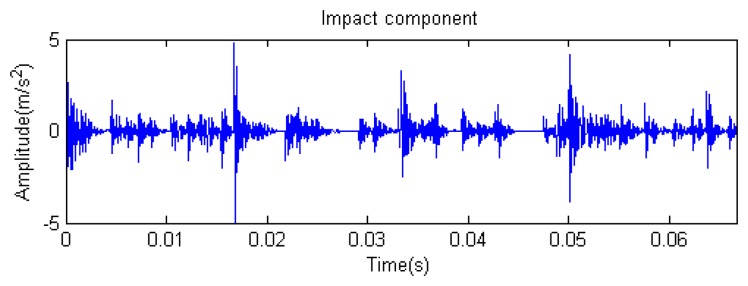
Impact component.

**Figure 5.**
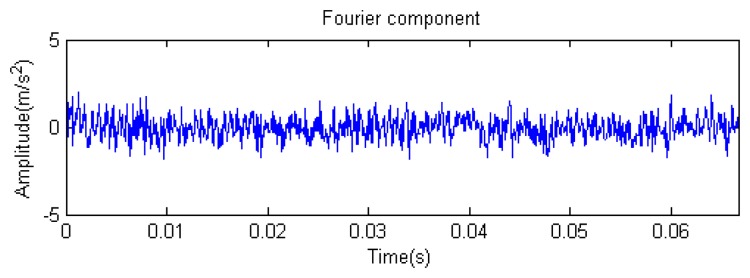
Fourier component.

**Figure 6.**
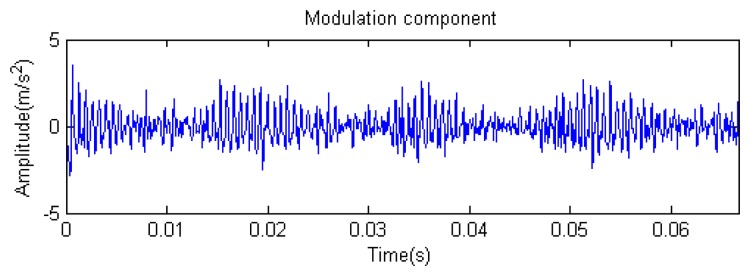
Modulation component.

**Figure 7.**
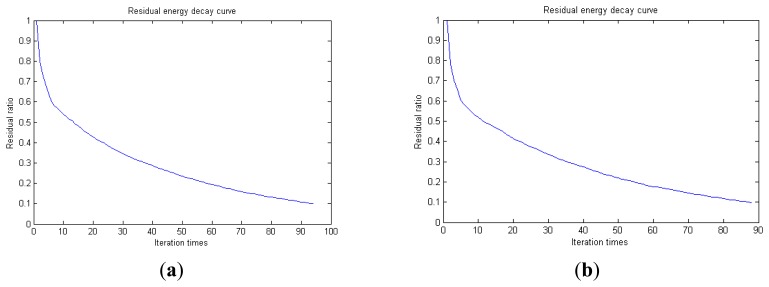
Residual energy decay curve: (**a**) first 512 points; (**b**) last 512 points.

Traditional termination conditions of iteration are usually set as the upper bound of the number of iterations or the energy of the residual signal less than a certain threshold. In this section, the termination condition of iteration is set to be the threshold of the energy ratio of the residual signal to the original signal (the residual ratio ≤ 0.1). The waveform and spectrum of the reconstructed signal are shown in Figure 3. Compared with Figure 2, the algorithm has clearly favorable reconstruction precision. Here, the signal is reconstructed only through each feature atom dictionary; Figures 4, 5 and 6 show the impulse component, Fourier component and modulation component, respectively. As the length of the basic atom is set to 512 points, so the signal to be analyzed is divided into two parts for matching pursuit decomposition and reconstruction. Figure 7 shows the curved residual ratio of the two parts.

In Figure 7, the numbers of iterations of these two parts are 93 and 87. That is to say, the analysis signal is expanded linearly on the 93 and 87 atoms in the over-completed composite dictionary. However, bad sparsity limits a lot; a large amount of noise components will be introduced, which causes the characteristic information of each component in Figures 4, 5 and 6 to be not so obvious. The residual signal energy is approximately exponential decay, and the amplitude attenuation of the first few orders is greater than those of the higher orders.

Define the original signal component in Figure 1 as the useful component, and the artificially added random signal component conforming to the normal distribution as the noise component. Then, analyze the effect about the energy ratio of the useful component with respect to the noise component of the signal. The signal-to-noise ratio (*SNR*) of the signal with noise was −0.7339 dB. The energy ratio of the useful components to noise components could be calculated as 0.4581. It could be thought that 45.81% of the energy of the signal with noise is useful signals, with the rest being noise. Thus, it is not scientific to set the termination conditions of the iteration as the energy ratio of the residual signal to the original signal *η* ≤ 0.1. In this condition, a large amount of the noise signals (90% − 45.81% = 44.19%) are also reconstructed.

#### 4.2.2. Improvement Termination Condition of Iteration

However, in the analysis of sensor-based vibration signals measured from an engineering gearbox, since the energy ratio of the noise in the signal is unknown in advance, a threshold of the energy ratio of the residual signal to the original signal cannot be determined. This is the major defect in using the threshold of the residual ratio as the termination condition of iteration. In [26], the disadvantage of this termination condition of iteration is discussed, and in [27], it has been proven that under the condition of the limited length of the signal, the energy of the residual signal would show exponential decay with the increase of the number of iterations. Here, the constructed function is *τ* = *e*^−^*^a^*^(^*^m^*^−1)^, where *a* is the attenuation coefficient. In the *m*-th matching process, if the energy ratio of the residual signal to the original signal satisfies Equation (9),

(9) $$\eta_{m}=\frac{E(m)}{E(1)}\leq e^{a(1-m)}$$

the matched signal components are considered as the useful components; otherwise, they are the noise components, and the iteration is terminated. The attenuation coefficient *a* is determined empirically. It could be confirmed that the greater the intensity of noise in the signals to be analyzed, the smaller the value of *a* should be.

**Figure 8.**
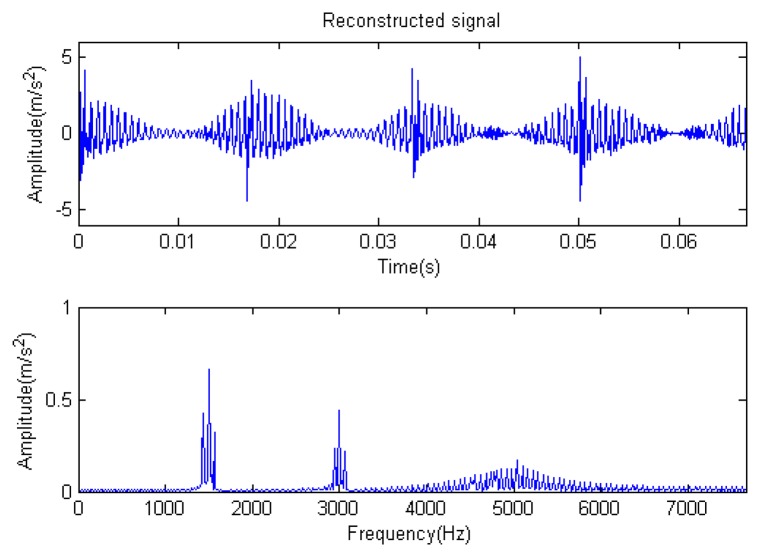
Reconstructed signal.

**Figure 9.**
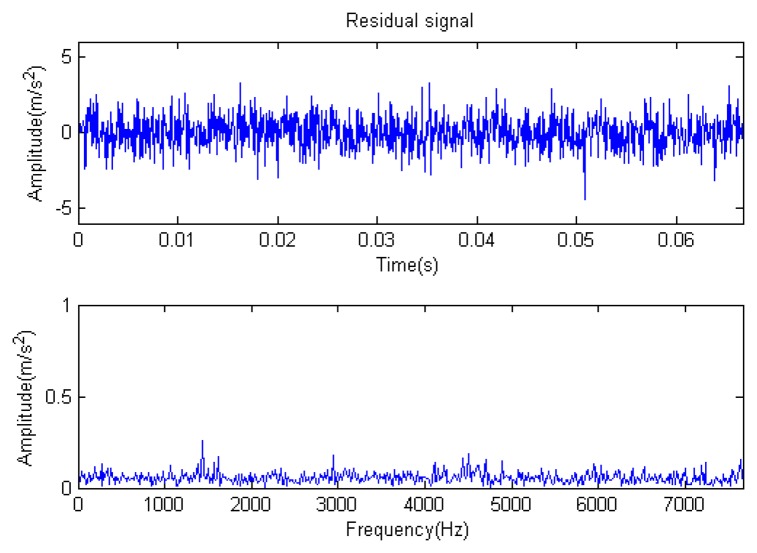
Residual signal.

**Figure 10.**
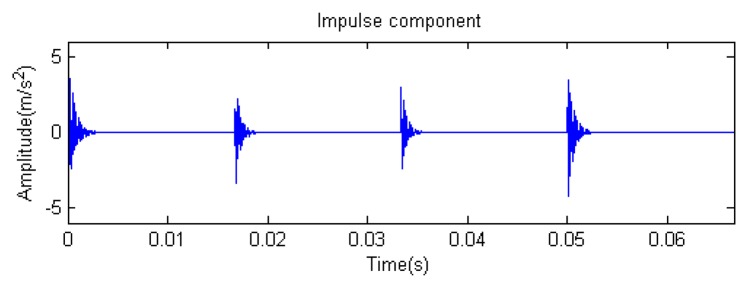
Impact component.

**Figure 11.**
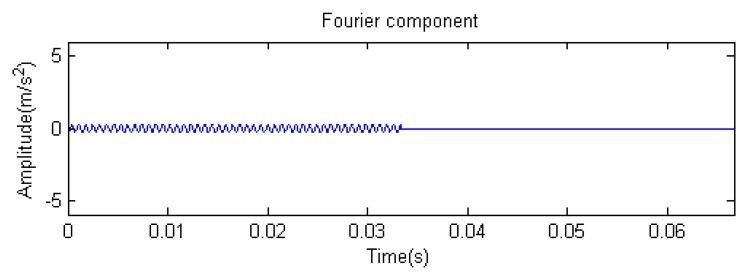
Fourier component.

**Figure 12.**
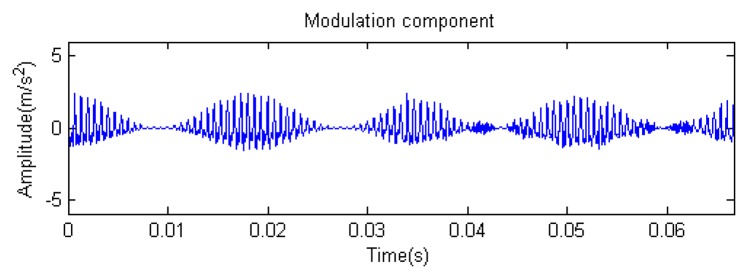
Modulation component.

The signal to be analyzed is still the simulation signal of the faulty gear with the noise mentioned before, as shown in Figure 2. The algorithm and parameters adopted in the analysis are consistent with those in Section 4.2.1, but the termination condition of iteration is changed. The termination condition of iteration based on the attenuation coefficient given in Equation (9) was adopted. The attenuation coefficient was *a* = 0.10. The reconstructed signal and residual signal are shown in Figures 8 and 9, respectively. Comparing the waveforms and spectra of Figures 1 and 8, the reconstructed signals are basically useful signal components, while the residual signals are basically noise components (the spectrum is a broad band with no obvious peak). Separate reconstructed impulse components, Fourier components and modulation components are shown in Figures 10, 11 and 12. The feature information is obvious, and the impulse components are represented as four evenly spaced impulses. Fourier components are represented as an obvious sine signal or no signal. Modulation components also present obvious modulation characteristics.

**Figure 13.**
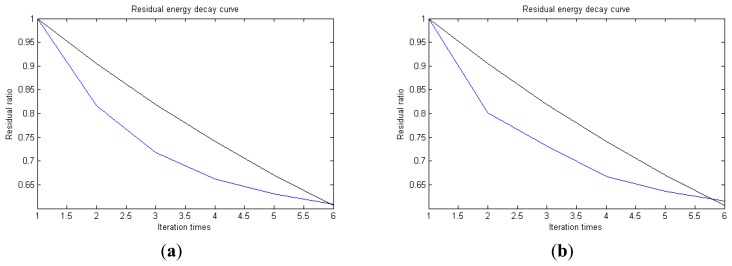
Residual energy decay curve: (**a**) first 512 points; (**b**) last 512 points.

In Figure 13, the residual ratio attenuation curves of the matching pursuit decomposition of the two parts of the signal are given. One line in the plot of Figure 13 stands for the residual energy decay curve, and the other stands for the change of *τ*, which was mentioned before. The lines in the figures below of the residual energy decay curve have the same meaning. It could be seen that when the termination condition of iteration based on the attenuation coefficient is adopted, the iteration numbers of these parts are all five. Compared with the attenuation coefficient of the residual ratio threshold, this condition greatly enhances the sparsity of decomposition. Furthermore, the energy ratio of each component of the reconstructed signal could be calculated. It could be seen from the residual ratio attenuation graph that at the end of iteration, about (39.1% + 38.4%)/2 = 38.75% of the signal components are extracted, which is very close to the 45.81% useful signal components. Then, it could be concluded that most (38.75%/45.81% = 84.59%) of the useful signal components are extracted, containing nearly no noise.

#### 4.2.3. Comparison of the Results

Table 1 gives the comparison results. Obviously, the termination condition of iteration based on attenuation coefficient is superior to that based on the residual ratio threshold in terms of the effect of matching pursuit.

Theoretical analysis: the setting of the termination condition of iteration based on the attenuation coefficient is the same as threshold processing, both having a noise reduction effect. The difference is that the threshold-based procedure reduces noise in the reconstruction process, while the attenuation coefficient-based procedure avoids the pollution of the noise signal in the process of decomposition, thus greatly reducing the computational complexity and enhancing the sparsity of decomposition.

### 4.3. Comparison of Different Feature Atom Libraries

#### 4.3.1. Fourier Dictionary and Impulse Time-Frequency Dictionary

**Figure 14.**
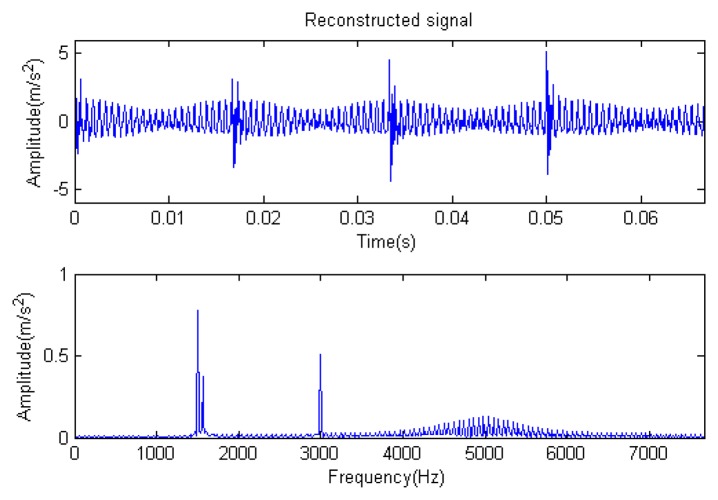
Reconstructed signal.

**Figure 15.**
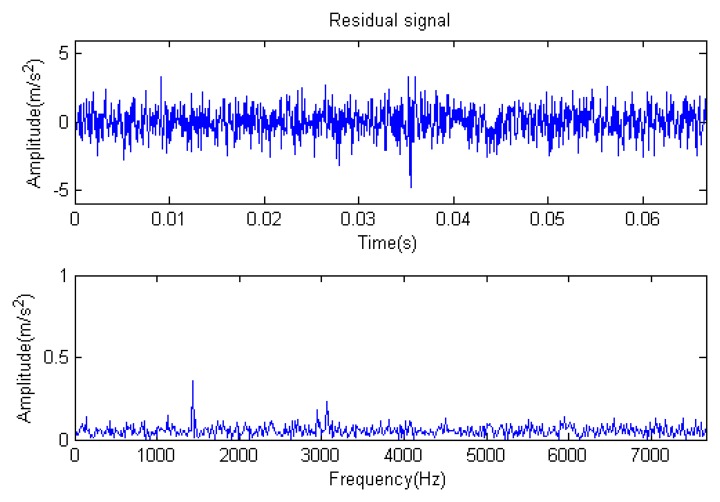
Residual signal.

**Figure 16.**
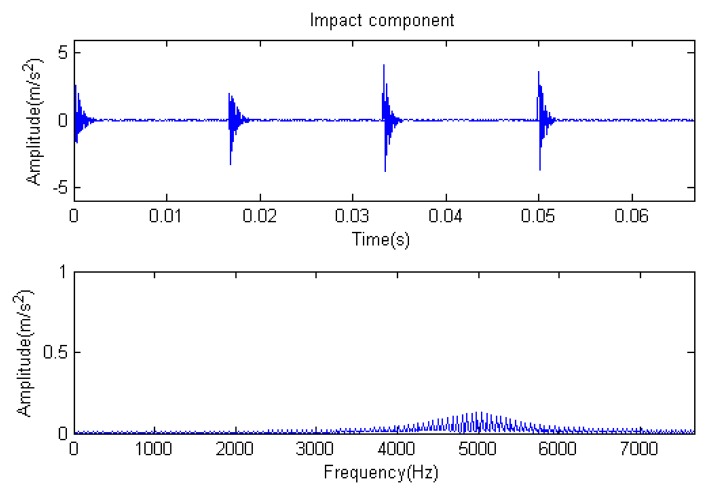
Impact component.

**Figure 17.**
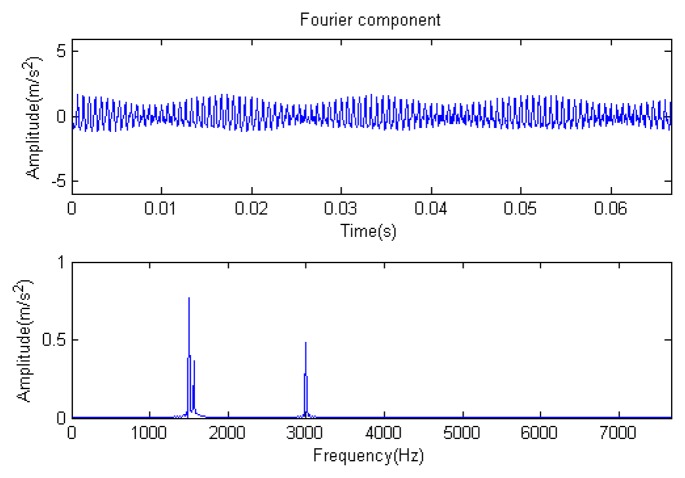
Fourier component.

The signal to be processed is still the simulation signal of faulty gear with noise with a length of 1024. The CD-SaMP algorithm is applied. The composite dictionary is composed of the Fourier feature atom library and impulse feature atom library. The termination condition based on the attenuation coefficient is adopted, with the attenuation coefficient set as *a* = 0.1. The SNR of the signal to be analyzed is −0.8532 dB, with the energy of useful components accounting for 45.94% of the total energy of the signal. The analysis results are as follows: Figures 14 and 15 are the reconstructed signal and the residual signal. The separated reconstructed impulse components and Fourier components are shown in Figure 16 and Figure 17, respectively. It could be seen that the feature signal reconstructed by the matching pursuit algorithm based on the attenuation coefficient is very obvious.

**Figure 18.**
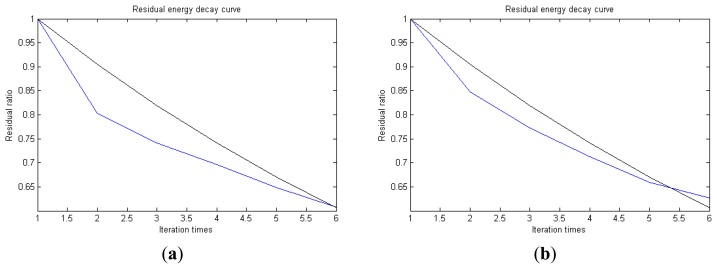
Residual energy decay curve: (**a**) first 512 points; (**b**) last 512 points.

In Figure 18, the residual ratio attenuation curves of the two parts are given. For the first 512 points, the iteration number is five. For the last 512 points, the iteration number is also five. Additionally, at the end of the iteration, the ratio of the energy of the extracted signal is (39% + 37%)/2 = 38%. The energy ratio of the known useful signal components is 45.94%. The extraction rate of the useful components is 38%/45.94% = 82.72%. It is reasonable to believe that most of the useful signal components have been extracted, with nearly no noise.

#### 4.3.2. Modulation Dictionary and Impulse Time-Frequency Dictionary

**Figure 19.**
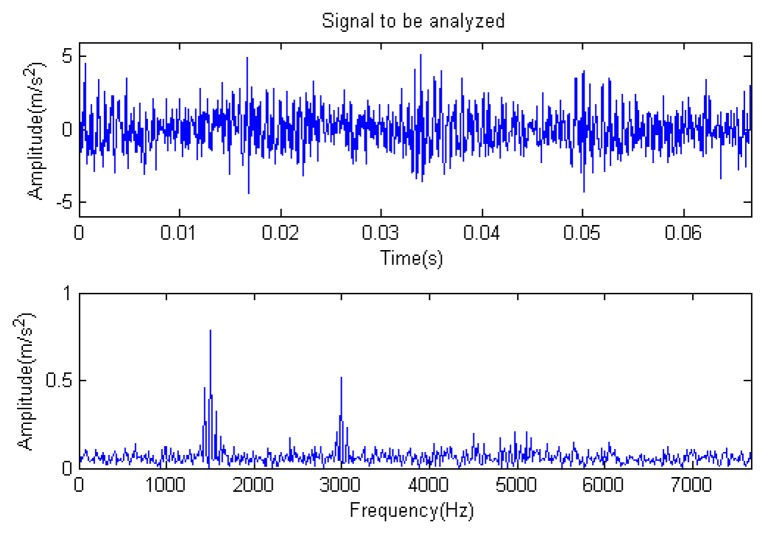
Signal to be analyzed.

**Figure 20.**
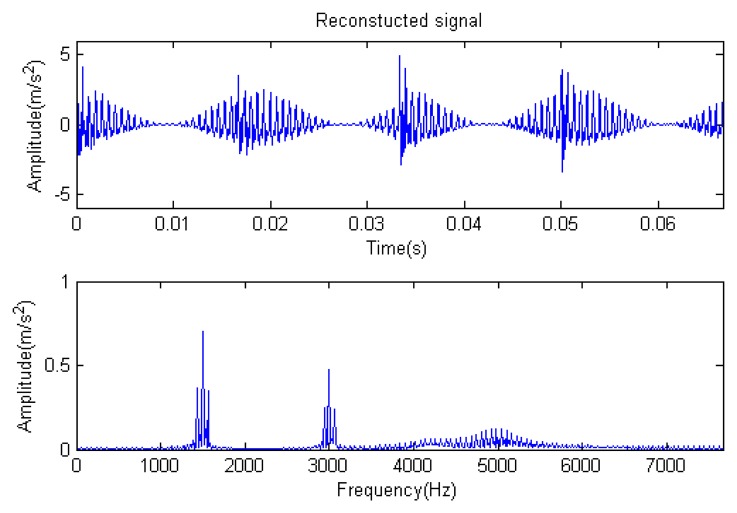
Reconstructed signal.

**Figure 21.**
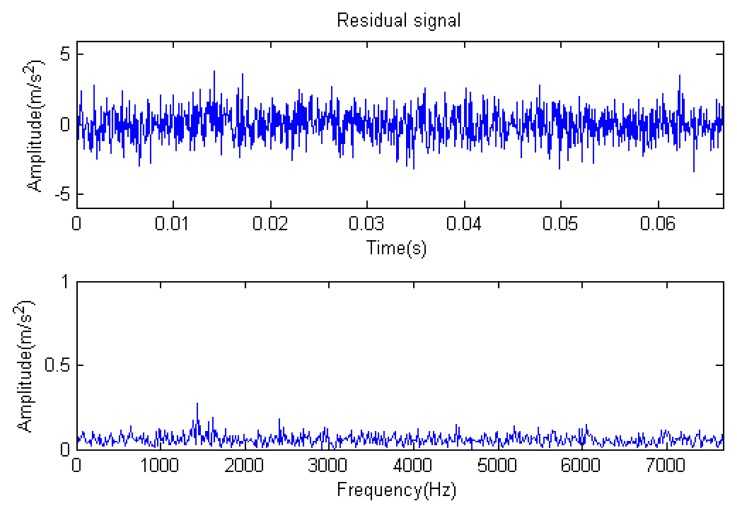
Residual signal.

**Figure 22.**
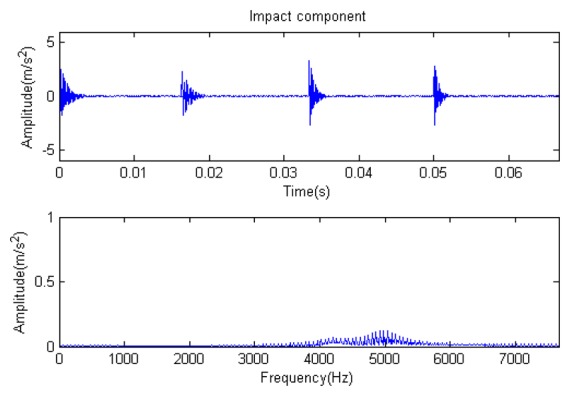
Impact component.

**Figure 23.**
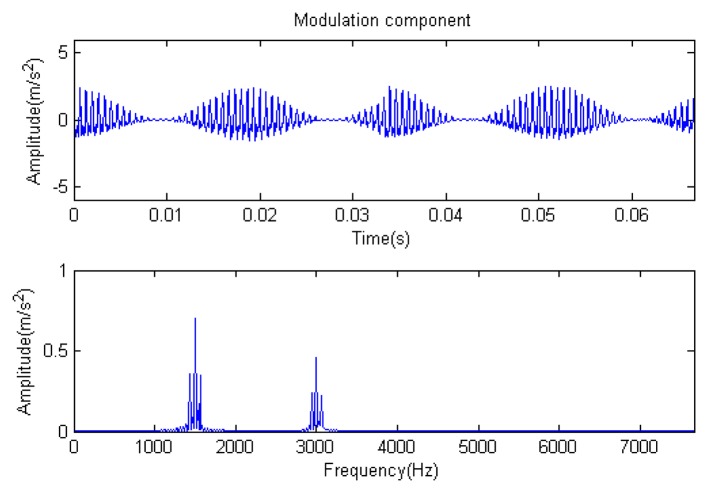
Modulation component.

The signal to be processed is still the simulation signal of the faulty gear with noise with a length of 1024. The CD-SaMP algorithm is applied. The composite dictionary is composed of the modulation feature atom library and impulse feature atom library. The termination condition based on the attenuation coefficient is adopted, with the attenuation coefficient set as *a* = 0.13. The *SNR* of the signal to be analyzed is −1.0153 dB, with the energy of useful components accounting for 43.84% of the total energy of the signal. The analysis results are as follows: Figures 19, 20 and 21 are the signal to be analyzed, the reconstructed signal and the residual signal. The separated reconstructed impulse components and modulation components are shown in Figure 22 and Figure 23, respectively. From this result, it could be presented that the feature information of the feature components is very obvious.

**Figure 24.**
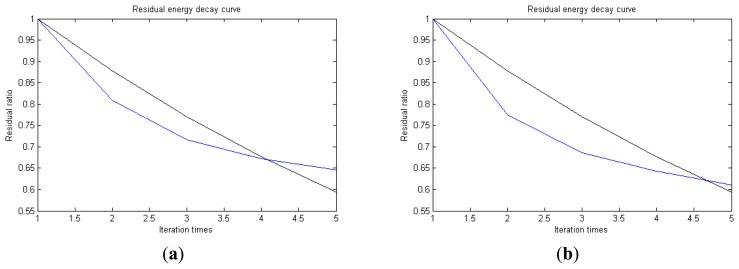
Residual energy decay curve: (**a**) first 512 points; (**b**) last 512 points.

In Figure 24, the residual ratio attenuation curves of the two parts are given. For the first 512 points, the iteration number is four. For the last 512 points, the iteration number is also four. Additionally, at the end of the iteration, the ratio of the energy of the extracted signal is (35% + 39%)/2 = 37%. The energy ratio of the known useful signal components is 43.84%. The extraction rate of the useful components is 37%/43.84% = 84.40%. It is reasonable to believe that most of the useful signal components have been extracted, with nearly no noise.

#### 4.3.3. Comparison of the Results

From Table 2, we can see that the attenuation coefficient *a* of the modulation dictionary is set higher, that is to say the degree of attenuation required is greater. The modulation dictionary extracts more useful components with less iterations. The modulation dictionary extracts more complete frequency components. The above analysis shows that in the matching analysis of the simulation signal of the faulty gear with noise, the modulation dictionary is superior to the Fourier dictionary.

## 5. Comparison of the Single-Atom and Multi-Atom Matching Based on the Improvement Termination Condition of Iteration

### 5.1. Composite Dictionary Multi-Atom Matching Pursuit

**Figure 25.**
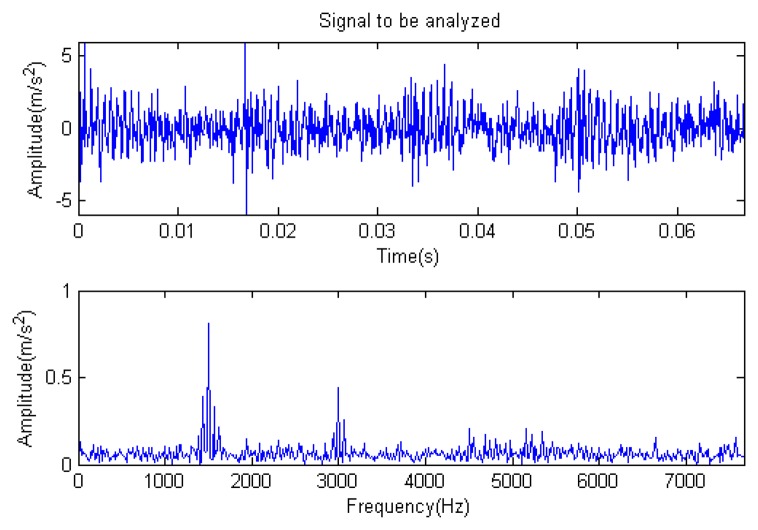
Signal to be analyzed.

**Figure 26.**
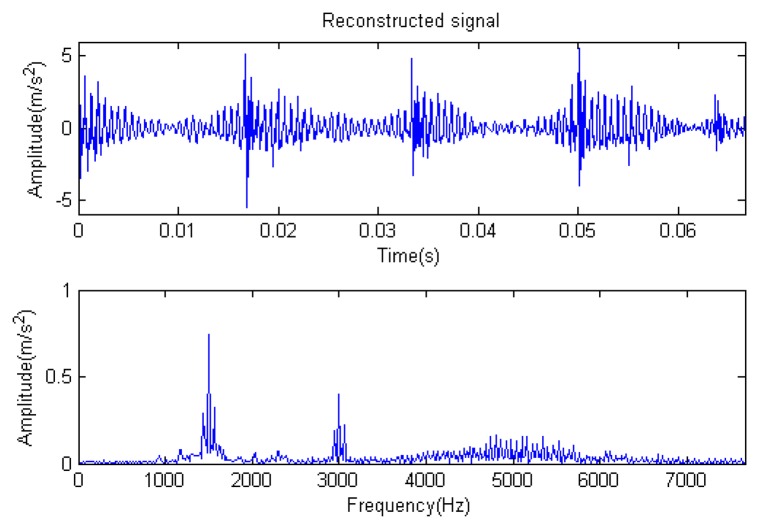
Reconstructed signal.

**Figure 27.**
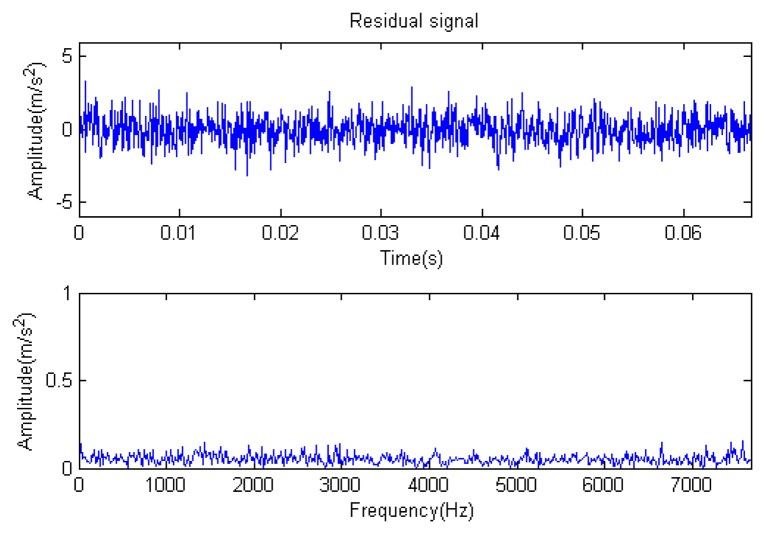
Residual signal.

**Figure 28.**
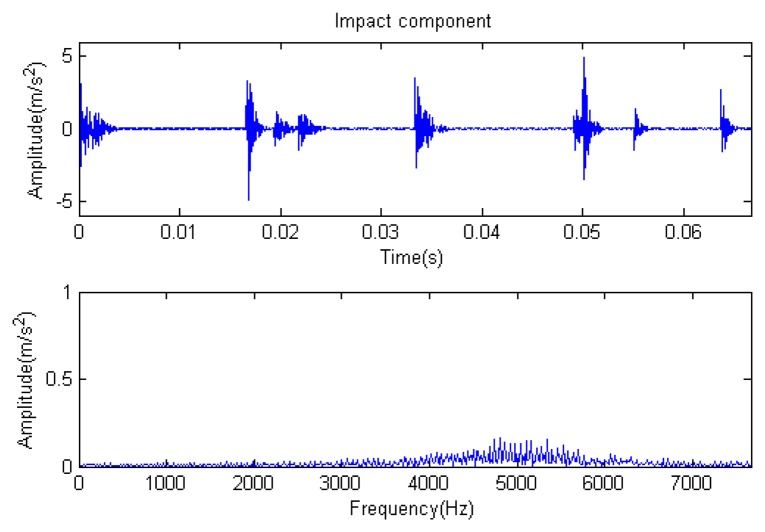
Impact component.

**Figure 29.**
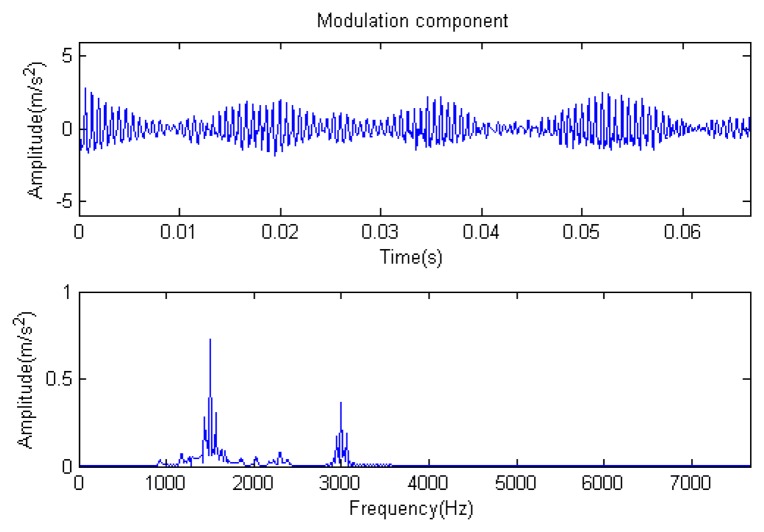
Modulation component.

The signal to be processed is still the simulation signal of the faulty gear with noise, the decomposition and reconstruction method adopts the algorithm mentioned in [19] as well as its parameter setting in order to get a clear comparison. Since the parameter setting in GA affects a lot, so setting Φ = 0 simplifies the problem. A certain scale of population ensures the effectiveness of finding the best matching atom. Because of more parameters in the time-frequency dictionary, the population size is set to be 300, while the modulation dictionary sets 200 according to its two parameters. The maximal number of evolution generations is 100; the single-point mode is adopted with 0.6 as the crossing probability and single-point mutation is adopted with a probability of 0.1. The termination condition based on the attenuation coefficient was adopted, with the attenuation coefficient set as *a* = 0.12. The signal-to-noise ratio (*SNR*) is −0.8241 dB, and the energy ratio of the known useful signal components is 45.04%. Figures 25, 26 and 27 are the signal to be analyzed, the reconstructed signal and the residual signal, respectively. The separated reconstructed impulse components and modulation components are shown in Figures 28 and 29, respectively.

**Figure 30.**
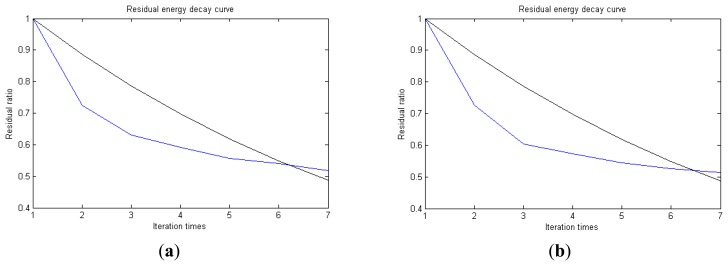
Residual energy decay curve: (**a**) first 512 points; (**b**) last 512 points.

Figure 30 gives the residual ratio attenuation curves of the two parts. For the first 512 points, the iteration number is six. For the last 512 points, the iteration number is also six. Additionally, at the end of the iteration, the ratio of the energy of the extracted signal is (48.19% + 48.69%)/2 = 48.44%. The energy ratio of the known useful signal components is 45.04%. The extraction rate of the useful components is 48.44%/45.04% = 107.55%. This is impossible, so the reconstructed signal includes a large amount of noise.

The Table 3 shows the matching results. For booth impulse atoms and modulation atoms, from the beginning of the third iteration, the matching coefficient amplitude decreased significantly slowly. Furthermore, the frequency of the modulation atoms was biased a lot by the ideal frequency components.

### 5.2. CD-SaMP Algorithm Based on the Improvement Termination Condition of Iteration

**Figure 31.**
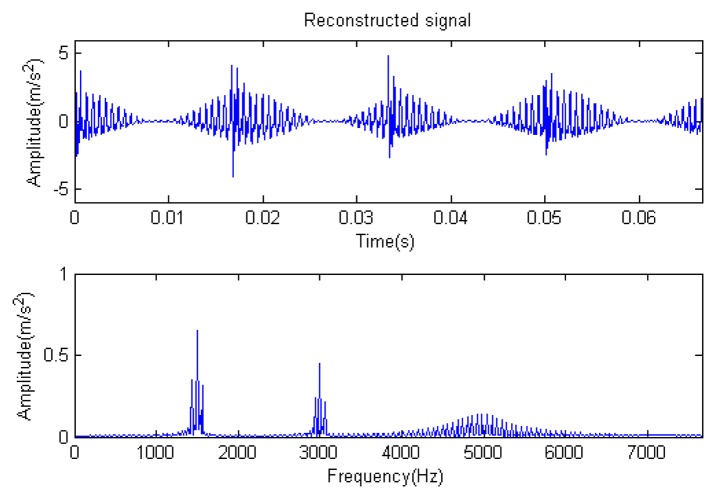
Reconstructed signal.

**Figure 32.**
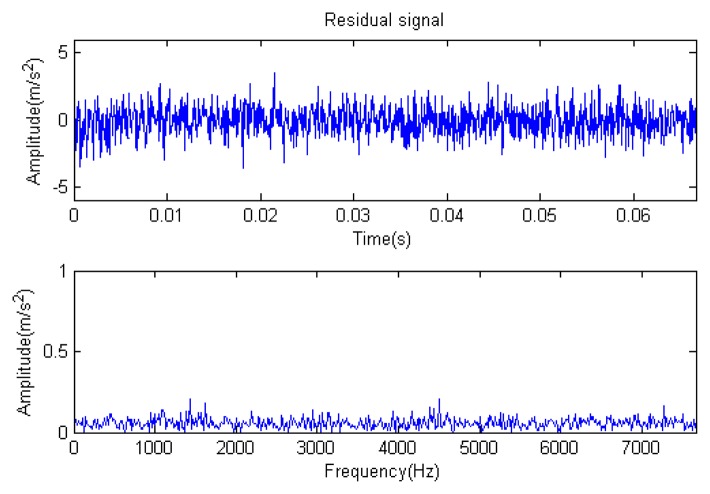
Residual signal.

**Figure 33.**
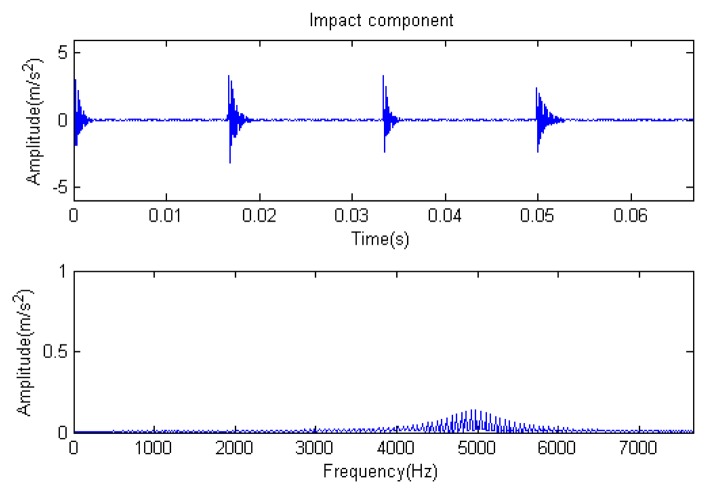
Impact component.

**Figure 34.**
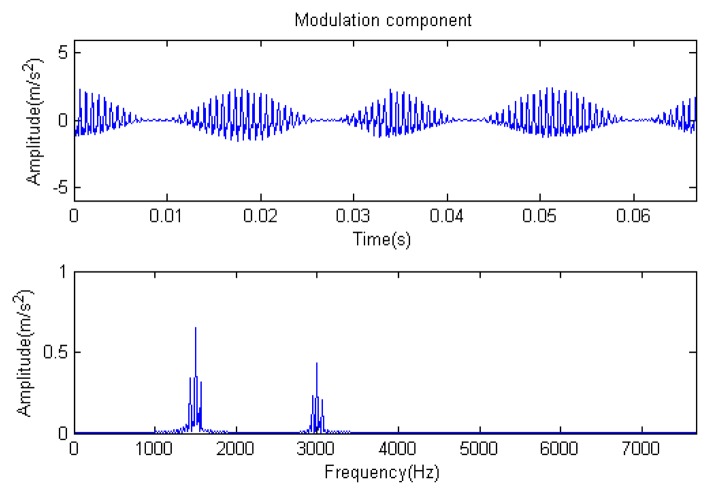
Modulation component.

The signal to be processed is still the simulation signal of the faulty gear with noise; the decomposition and reconstruction method adopts the CD-SaMP algorithm. The parameter settings are the same as in Section 5.1. The signal-to-noise ratio (*SNR*) is −0.9338 dB, and the energy ratio of the known useful signal components is 45.97%. Figures 31 and 32 are the reconstructed signal and the residual signal. The separated reconstructed impulse components and modulation components are shown in Figures 33 and 34, respectively. Compared with Figures 28 and 29, the reconstructed signal is pure with little noise, and the feature information is very obvious.

**Figure 35.**
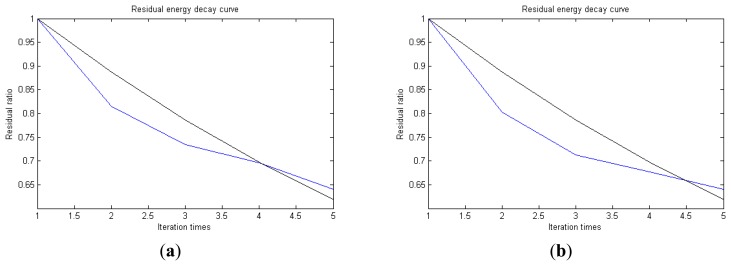
Residual energy decay curve: (**a**) first 512 points; (**b**) last 512 points.

Figure 35 gives the residual ratio attenuation curves of the two parts. For the first 512 points, the iteration number is four. For the last 512 points, the iteration number is also four. Additionally, at the end of the iteration, the ratio of the energy of the extracted signal is (35.92% + 35.91%)/2 = 35.915%. The energy ratio of the known useful signal components is 45.97%. The extraction rate of the useful components is 35.915%/45.97% = 78.13%. The matching results are shown in Table 4.

### 5.3. Comparison of CD-SaMP and Composite Dictionary Multi-Atom Matching Pursuit

Table 5 gives the result comparison of the CD-SaMP and composite dictionary multi-atom matching pursuit. When the attenuation coefficient is the same, *i.e.*, *a* = 0.12, the number of iterations of the single-atom matching is 4 + 4, but multi-atom matching is 6 + 6; the number of matching atoms of the single-atom matching is 4 + 4, but multi-atom matching is 12 + 12. From the decomposition from the sparsity point of view, the CD-SaMP is better than the composite dictionary multi-atom matching pursuit.

Although composite dictionary multi-atom matching pursuit extracts great energy from the effective components, at the same time, it introduces noise. From the beginning of the third iteration, the matching coefficient amplitude decreased significantly slowly. We can also see from the frequency information from the modulation atoms that the matching component may not be the useful component from the beginning of the third iteration, while the CD-SaMP extracts the effective components in 78.13%, with no noise.

Above all, the CD-SaMP is superior to that composite dictionary multi-atom matching pursuit in terms of the effect of matching pursuit.

Theoretical analysis: in composite dictionary multi-atom matching pursuit, every iteration process will search for a multi-atom: an impulse atom and a modulation atom; while CD-SaMP only finds the perfect atom, either the impulse atom or the modulation atom. From the point of view of the gear fault simulation signal component, the impact component energy is less than the modulation components. However, the greedy principle of matching pursuit is always a priority matching maximum energy ratio of the components, which explains why the CD-SaMP gets two modulation atoms first and two impulse atoms next. However, each step of the composite dictionary multi-atom matching pursuit will find a multi-atom with an impulse atom and a modulation atom, which is the total projection residuals. However, the total projection may not be the maximum energy ratio of the current residual signal. Therefore, from this point of view, the composite dictionary multi-atoms matching violates the principle of greedy matching pursuit.

## 6. Analysis of the Engineering Signal

**Figure 36.**
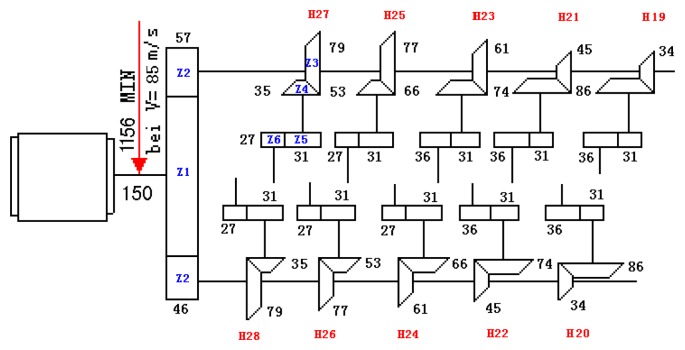
Driving chain of a gearbox for a high-speed finishing mill.

Figure 36 shows the driving chain of a gearbox on a high-speed finishing mill from a steel plant, this system has 10 vibration sensor measurement points for the status detection of the transmission system. The parameters of every component in the system are known. On-spot monitoring information indicated that modulation information had been reflected by the frequency spectrogram about the vibration data since 14 September 2006. The modulation frequency was the rotation frequency of Shaft II on the bevel box of finishing Support 22 (30.1 Hz) [19]. This phenomenon remained in later periods.

**Figure 37.**
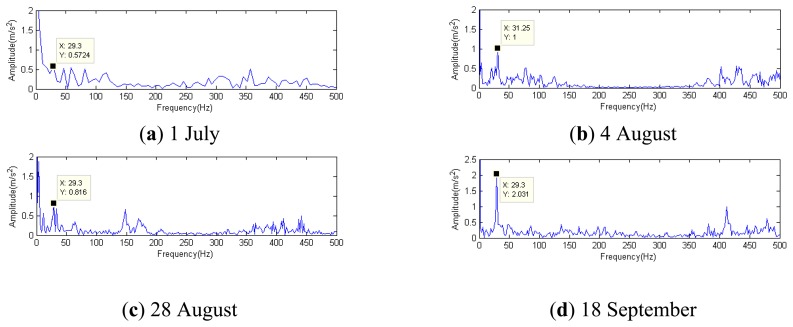
Demodulation spectra with composite dictionary single-atom matching.

In this paper, the CD-SaMP performs well in the simulation sensor-based vibration signal. Analyzing the historical data on 1 July, 4 August, 28 August and 18 September, their demodulation spectra are shown in Figure 37. The composite dictionary is built up with the impulse dictionary, modulation dictionary and Fourier dictionary. Choose the termination condition based on the iterative attenuation coefficient, and define *a* = 0.08, because of the complex engineering data with great noise.

**Figure 38.**
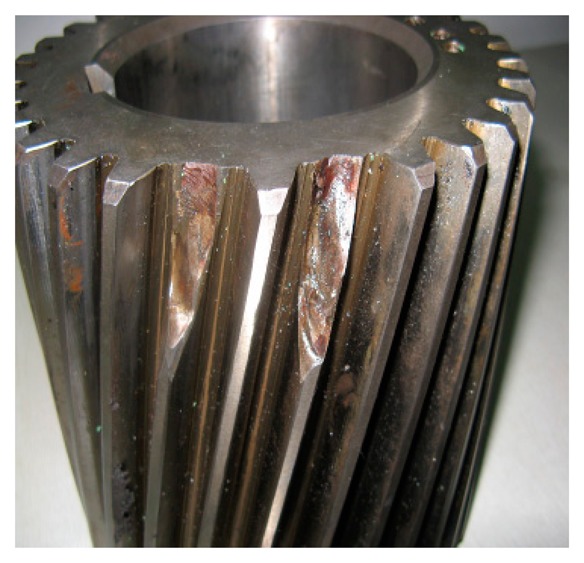
Collision of the gear Z5 on Shaft II of the bevel box.

Figure 37 clearly shows that the fault characteristic frequencies at 29.3 Hz or 31.25 Hz (there was certain deviation from the actual fault characteristic frequency of 30.1 Hz due to the frequency resolution) could be seen. Besides, the amplitude has an increasing trend at 0.5724, 1, 0.816 and 2.031. On 1 July, the fault characteristic frequency at 29.3 Hz was not so clear, with a general amplitude value. However, from the later data from 4 August, the amplitudes of the characteristic frequency performed a growth trend, which indicates that the gear faults on Shaft II of the bevel box have become prominent and had developed since 4 August or even earlier. The managers checked the dismantled finishing Support 22 in November and noticed that the gear Z5 (with 31 teeth) on Shaft II of the bevel box was broken, as shown in Figure 38.

## 7. Conclusions

Sparse decomposition based on matching pursuit is an adaptive sparse representation of signals. MP is a classic algorithm for sparse decomposition. However, the single atom dictionary and the enormous amount of computation limit much of the sparse expression of complicated and non-stationary sensor-based vibration signals. At the same time, in order to enhance and optimize the sparsity and application effect of the algorithm, a new CD-SaMP algorithm is proposed in this paper. The algorithm introduced the termination condition based on the attenuation coefficient to strengthen the sparsity of the decomposition and the noise reduction effect, which has a good effect in the extraction of the impulse signals of the faulty gear. The influence of the selection of the feature atom library on the matching effect is also discussed here. The simulation analysis results show that the atom library with the modulation feature has a better matching effect than the atom library with the Fourier feature. The algorithm above was later applied to the analysis of the engineering signals of the faulty gear, which shows the feasibility and effectiveness of the proposed algorithm. Further research is currently undergoing in the quantitative diagnosis of sensor-based gearbox signals and for improving the proposed method to make it more feasible.

## Figures and Tables

**Table 1.** Comparison results of different termination conditions of iteration.

Items of Comparison	Termination Condition	Termination Condition
	Based on the Threshold of Residual Ratio	Based on the Attenuation Coefficient
Sparsity of decomposition	Iteration of (93 + 87) times, (sparsity not good)	Iteration of (5 + 5) times, (sparsity good)

Proportion of the useful signal component	90%/45.81% = 196.46% (more than 100%, impossible)	84.59%

Features of each component in the reconstructed signal	Not obvious	Very obvious
Whether the reconstructed signal is polluted with noise	Very strong	Almost no noise

**Table 2.** Comparison of the results.

Comparison Items	Fourier Feature Atom Library	Modulation Feature Atom Library
Attenuation coefficient (*a*)	0.10	0.13
Iteration times	5 + 5	4 + 4
Extraction rate of useful ingredients	82.72%	84.40%
Extracted frequency components (approximation, Hz)	1500, 3000, 1560	1500, 1500 ± 60, 3000, 3000 ± 60

**Table 3.** Matching results.

Number of Iterations		1	2	3	4	5	6
First 512 points	Matching coefficient (Impulse atom)	9.0255	6.7869	3.5555	3.5401	2.7563	3.3172

	Matching coefficient (Modulation atom)	13.7303	6.8350	4.9094	4.5045	−2.9403	−3.0417

	Frequency of Modulation atom (Hz)	*f*_1_ = 52,	*f*_1_ = 55,	*f*_1_ = 158,	*f*_1_ = 79,	*f*_1_ = 248,	*f*_1_ = 107,
		*f*_2_ = 1503	*f*_2_ = 3002	*f*_2_ = 1466	*f*_2_ = 1571	*f*_2_ = 1174	*f*_2_ = 1446

Last 512 points	Matching coefficient (Impulse atom)	7.6713	6.8782	4.2593	3.2684	2.5153	2.6608

	Matching coefficient (Modulation atom)	14.1232	8.3251	3.2998	−3.7668	−3.3469	2.2177

	Frequency of Modulation atom (Hz)	*f*_1_ = 52,	*f*_1_ = 52,	*f*_1_ = 75,	*f*_1_ = 203,	*f*_1_ = 126,	*f*_1_ = 145,
		*f*_2_ = 1501	*f*_2_ = 3004	*f*_2_ = 2301	*f*_2_ = 1643	*f*_2_ = 1385	*f*_2_ = 2032

**Table 4.** Matching results.

Number of Iterations		1	2	3	4
First 512 points	Matching coefficient	13.0852	8.6488	6.0016	7.1697

	Kind of Matching Atoms	Modulation	Modulation	Impulse	Impulse

	Frequency of Modulation atoms (Hz)	*f*_1_ = 55,	*f*_1_ = 56,		
		*f*_2_ = 1504	*f*_2_ = 3002	/	/

Last 512 points	Matching coefficient	13.3404	8.9558	5.7181	5.6537

	Kind of Matching Atoms	Modulation	Modulation	Impulse	Impulse

	Frequency of Modulation atoms (Hz)	*f*_1_ = 55,	*f*_1_ = 56,		
		*f*_2_ = 1502	*f*_2_ = 3003	/	/

**Table 5.** Comparison results.

Comparison Project	Single-Atom Matching	Multi-Atom Matching
The attenuation coefficient	0.12	0.12
Number of iterations	4 + 4	6 + 6
Number of matching atoms	4 + 4	12 + 12
Rate of extracting useful component	78.13%	78.46%
Extraction of frequency components (Approximate, Hz)	1500, 1500 ± 60, 3000, 3000 ± 60	1500, 1500 ± 60, 3000, 3000 ± 60
